# Long-lived coherences for the observation of oxidation kinetics on different timescales by NMR

**DOI:** 10.1038/s42004-026-02002-w

**Published:** 2026-04-16

**Authors:** Aude Sadet, Cristina Stavarache, Ioana Fidel, Mihai A. Voda, Andrei Ciumeica, Elena Ionita, Mihai Ciubotaru, Paul R. Vasos

**Affiliations:** 1 Biophysics and Biomedical Applications Group and Laboratory, Laser Gamma Experiments Department, LGED, Extreme Light Infrastructure, ELI-NP, “Horia Hulubei” National Institute for Physics and Nuclear Engineering IFIN-HH, Bucharest, Romania; 2 Faculty of Interdisciplinary Studies, ISDS, University of Bucharest, Bucharest, Romania; 3 Institute for Organic and Supramolecular Chemistry, ICOS, Bucharest, Romania; 4 Department of Life and Environmental Sciences, “Horia Hulubei” National Institute for Physics and Nuclear Engineering IFIN-HH, Bucharest-Măgurele, Romania; 5 Department of Immunology and Internal Medicine, Colentina Clinical Hospital, Bucharest, Romania

**Keywords:** Biophysical chemistry, NMR spectroscopy

## Abstract

For non-invasive imaging of biochemical processes, the ability of NMR to follow biomolecular transformations on different time windows needs to be improved. Long-lived coherences (LLC’s) are molecular-symmetry-adapted nuclear spin coherences that can be sustained for durations up to an order of magnitude longer compared to classical coherences in high-field magnets. 2D-LLC experiments provide contrast for targeted molecules against cell background, as demonstrated herein for glutathione (GSH), a major antioxidant in immune cells and rapidly-proliferating tumors. We tested the oxidative response of immune B-cells to exogenous incubated glutathione and high-dose-rate laser-driven radiation. We found agreement between biochemical methods and 2D-LLC for detecting GSH production induced by incubation. To also address oxidation on FLASH radiation response-relevant timescales, we demonstrate a direct 1D method, WINDOW-LLC. Using this method, we recorded multiple points of GSH oxidation kinetics within 15 seconds after a single excitation step. Thus, LLC’s find potential applications to detect oxido-reduction processes.

## Abstract (web summary)

The ability of nuclear magnetic resonance to probe biomolecular transformations on different time windows is essential for the non-invasive imaging of chemical and biochemical processes. Here, the authors show that long-lived coherences (LLC's) can facilitate the detection of biochemical processes on fast timescales and tracking of slow metabolic transformations in cells by using two-dimensional LLC to quantify the overexpression of glutathione (GSH) by immune B cells, and demonstrating a one-dimensional LLC method for recording multiple points of GSH oxidation kinetics within 15 seconds after a single excitation step.

## Introduction

Biochemical transformations occurring in cells on different timescales due to external stimuli such as oxidative agents, ionizing radiation, or chemotherapeutic agents can be followed non-invasively by magnetic resonance^1,2^. The cellular response to external factors amplifies the initial biochemical reactions, building up effects on metabolite networks within time intervals ranging from seconds to hours. Numerous spectroscopic techniques enable the in vivo or ex vivo detection of such modifications, both quantitatively and qualitatively, through the use of specific biomarkers^3^. Nuclear Magnetic Resonance (NMR) spectroscopy has the advantage of allowing the simultaneous observation of multiple metabolites without altering the sample or inducing structural modifications^4^. The nuclear spins of hydrogen-1, carbon-13, and nitrogen-15 nuclei are most often detected, yet the latter two require isotope enrichment. Chemically-related metabolites, such as pairs of oxidized/reduced species, can be detected to sense oxidation reactions. This is the case of glutathione (GSH), the most abundant cellular antioxidant in many cell types, especially fast-proliferating and immune B cells^5^. Its function is to maintain redox balance against reactive oxygen species (ROS) such as free radicals and peroxides that affect cell function. The kinetic equilibrium of GSH with its oxidized form, GSSG, can provide a functional biomarker for tissue response to therapy involving reactive molecular species, reflecting the cell’s response to oxidative stress^6^. GSH-dependent oxidation processes are central to radiation therapy delivered using high dose-rates. The biological effects of such high-dose-rate therapy are known as “FLASH”^7–10^: toxic side effects are minimized while ensuring tumor suppression. Understanding the dose-rate dependent mechanisms of radiation on biological systems involving free-radical formation and recombination can lead to significant improvements in radiotherapy^5,11^. In response to most types of naturally-occurring oxidative stress, the time scale of GSH oxidation kinetics in cells is hours; however, under FLASH-effect producing dose rates, which are on the order of 40–100 Gy/s, kinetics are accelerated, yielding high turnovers of oxidized GSH within seconds^3^. The oxidation of GSH by radiation-induced radicals can be quantified using, e.g., optical spectroscopy^12^ or NMR^13^, the latter being better adapted for translation to in-vivo detection of antioxidants within inner organs. The evolution of fast oxidation kinetics of GSH/GSSG species monitored by ^1^H NMR in solution yielded the rate constants *k* = *k*_uncat_ + *k*_cat_ [NaI] with *k*_uncat_ = 0.00045 mM∙min^−1^, and catalyzed reactions NaI accelerating the oxidation^13^ up to *k*_cat_ = 0.85 ± 0.05 min^−1^. In general, magnetic resonance observations cover timescales ranging from nanoseconds to hours^14^. However, limited phase memory and necessary magnetization recovery times of collective spin ensembles obscure some important observation timescales in both EPR^15^ and NMR^16^. Phase memory depends on molecular symmetry, on the gyromagnetic constant of detected nuclei^17^, and on the observed spin order. Spin order can be optimized for the given molecular substrate to extend the lifetime of its observation. For real-time NMR observations based on ^1^H nuclei, the timescale of several seconds is difficult to grasp, as it is too long compared to the relaxation time constants of standard transverse coherences and too short to allow repeated recoveries of longitudinal magnetization. In modern NMR, these processes often need to be studied via one excitation followed by multiple detections^13^, either because the initial excitation signal has been sensitivity-enhanced in an external site by dissolution-Dynamic Nuclear Polarization^18,19^ or because repeated excitations would extend experimental time beyond the targeted window. Long-lived coherences (LLCs), first introduced in high magnetic fields in 2010^20^, are a form of detectable transverse spin order featuring enhanced transverse relaxation time constants. In low magnetic fields, low-frequency oscillations with extended lifetimes for similar types of spin-order were introduced in 2009^21^. LLCs mainly minimize the impact of internal dipolar interactions and, to a small degree, the impact of external interactions^22–24^. Due to this, they allow improvements in signal linewidths by up to an order of magnitude compared to standard coherences, extending observation windows from hundreds of milliseconds to seconds in proteins and from below the second to several seconds in small molecules^20,24^. This can enable real-time observation of biochemical transformations, chemical exchange or diffusion processes that occur on this timescale. Using standard single-spin order, Ernst-angle^25^, or generally small-angle excitations can be used to extend the detection time domain, albeit at the cost of sensitivity. In the field of long-lived spin order, detection in successive steps and filtering can be achieved via small-angle pulses^13^ or especially-designed pulses as pioneered in ref. ^26^. However, whenever the full signal is to be detected for each step, as well as in the presence of inhomogeneous or difficult-to-reproduce excitation fields *B*_1_, direct repeated recording without any detection pulses is necessary. For LLCs, this can be achieved by the 1D sequence introduced herein, called WINDOW-LLC. Long-lived spin order can be sustained in the presence of *B*_0_ magnetic field inhomogeneities, as shown by prior studies where the inequivalence of the spin pairs could be masked by the sustaining field even in the presence of shifts from the carrier of almost the same order of magnitude as the sustaining field amplitude^27^. Variations in the sustaining field of up to 20% were also found herein to be well tolerated (Supplementary Note 1), provided the amplitude of the field is above 3 kHz.

Following the molecular kinetics of oxidative processes by NMR involves establishing distinct observables for reduced and oxidized biomarkers. The oxidized and reduced forms of antioxidants are structurally close, leading to spectral similarities. To address the spectral challenges introduced by the frequency proximity between detected species and with other metabolites, several strategies have been developed to improve resolution, such as reducing signal overlap by isotopic labeling^28,29^ and two-field NMR^30^, reducing enhancing relaxation times and reducing line widths by multi-dimensional NMR techniques^31–36^. Enhanced relaxation times also create contrast against the background, and this can be used to selectively detect slowly-relaxing spin groups, such as protein parts featuring fast motions^37,38^. In this work, we propose long-lived coherences (LLCs) to obtain contrast against the cell background for glutathione^20,23,24,39^. For in-cell ^1^H NMR studies, resolution is limited by the coherent and incoherent relaxation mechanisms, and signal overlaps and line broadening hurdle biomarker assignment. LLCs offer a powerful tool for analyzing metabolite mixtures, significantly reducing signal overlap and enhancing the assignment and characterization of individual components, improving the clarity of spectral data^23^. The effectiveness of LLCs in complex systems outperforms traditional relaxation-editing methods that rely on *T*_1_ and *T*_2_ relaxation time constants^40^.

As demonstrated in the seminal 2010 article^20^, LLCs can be initialized in high magnetic fields in couples of diastereotopic aliphatic protons via a straightforward coherent superposition of transverse operators with opposing signs on the two *J*-coupled proton spins, I and S: ${\rho }_{\mathrm{LLC}}^{0}={I}_{x}-{S}_{x}$. LLCs correspond, in terms of singlet and triplet states, to coherent superpositions of nuclear spin states of singlet (*S*_0_) and central triplet (T_0_). For a spin-1/2 pair, the singlet state is ${S}_{0}=\frac{1}{\sqrt{2}}(|{\alpha }_{I}{\beta }_{S}\rangle -|{\beta }_{I}{\alpha }_{S}\rangle )$ and the central triplet state is ${T}_{0}=\frac{1}{\sqrt{2}}(|{\alpha }_{I}{\beta }_{S}\rangle +|{\beta }_{I}{\alpha }_{S}\rangle )$, where *α*_*I*, *S*_ and β_*I*, *S*_ represent the spin-up and spin-down states of each of the two spins, which are rendered equivalent by radio-frequency sustaining to adopt eigenstates that are characteristic for low magnetic fields^21,41^.

Recently, LLC excitation was rendered possible also beyond proton pairs that are isolated from *J*-couplings with external spins, i.e., in aliphatic chains with ranges of neighboring methylene sites, a significant improvement that allows the study of a larger class of molecules^42^. For glutathione, an isolated pair of protons is available at the level of the Gly residue, (*I*, *S*) = Gly-(H^α2^, H^α3^), and the original LLC method was used for excitation^20^, with the notable improvement of using “SLIC”-based transfer^43^.

As NMR and MRI evolve towards functional molecular diagnostic^19^, we present herein a way of following, by real-time NMR, the signals of GSH/GSSG, the main cellular redox pair in fast-proliferating cells, in order to study cellular oxidation processes. LLCs are used both for multi-dimensional experiments for the monitoring of slow redox transformations and for following fast oxidation kinetics in real time. WINDOW-LLC directly detects LLCs in 1D experiments for the glutathione redox pair (GSH/GSSG) over time scales of tens of seconds. The WINDOW-LLC method could also be used in the future in experiments combining LLC detection with signal enhancement via dissolution-based Dynamic Nuclear Polarization (DNP)^18^ for following oxidation reactions. Moreover, the windowed sustaining of LLCs reduces the average delivered radio-frequency power, as detailed in prior work^23^. Extending signal lifetimes is essential^22,44,45^ for signal enhancement for both in-cell and in-vivo applications. ^1^H-based LLC spectroscopy is thus developed in order to be used, together with techniques that involve labeling molecular substrates with carbon-13 or nitrogen-15 isotopes, for the study of oxidation kinetics on both fast and slow timescales.

## Results and discussion

**Fig. 1:**
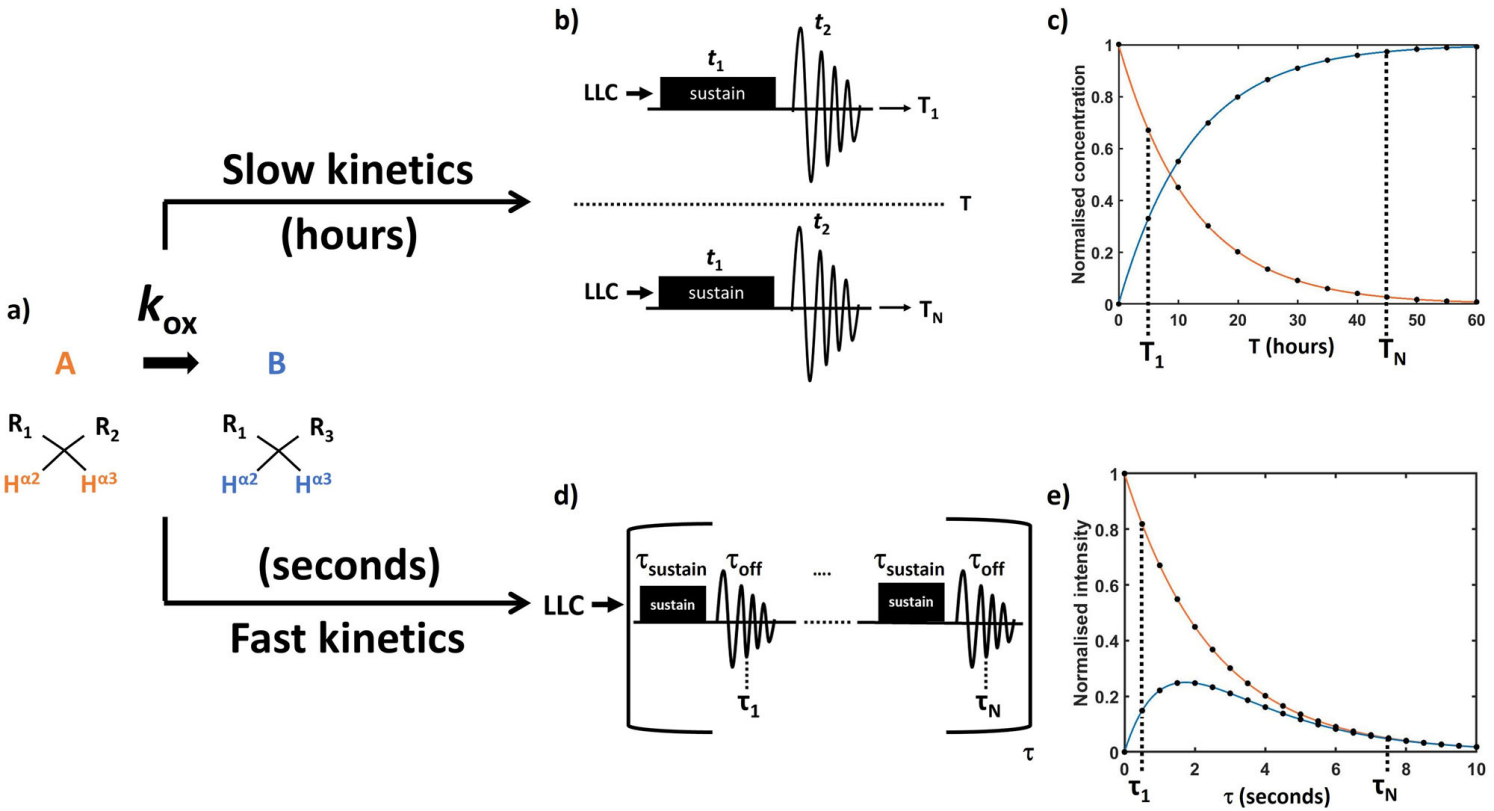
Oxidation reactions studied via long-lived coherences (LLC). **a** Representation of a chemical reaction transforming biomarker A into biomarker B. Both biomarkers A and B feature two diastereotopic protons (H^α2^, H^α3^) adapted for sustaining LLCs. **b** 2D LLC spectroscopy to obtain spectral intensities from the two molecular forms A, B at different times during an oxidation reaction on the timescale of hours. **c** Spectral intensities are plotted to extract kinetics of NMR-detected concentrations of molecules transformed by the reaction, e.g., the conversion from the reduced form (orange curve) to oxidized form (blue curve). **d** 1D WINDOW-LLC pulse sequence designed to obtain molecular NMR spectral intensities at different times during chemical reaction, on the timescale of seconds. LLCs are created via a 90° radio-frequency pulse followed by a SLIC radio-frequency irradiation^43^ applied with an amplitude equal to the *J*-coupling between the two protons Gly (H^α2^, H^α3^). LLCs are then sustained by a radio-frequency field. The sustaining field *B*_1_ is applied intermittently during the LLC evolution period, being activated during delays of ${\tau }_{\mathrm{sustain}}$ and deactivated during ${\tau }_{\mathrm{off}}$ (detailed in “Materials and Methods”) in order to open the receiver for detection. **e** Detection of the two molecular forms on the time scale of seconds using the 1D direct method. Transverse relaxation (phase memory loss) limits the upper observation timescale to circa five times the relaxation time constant of LLCs. The initial spin order is not renewed during observation, at variance with the 2D-LLC method.

The methods used herein to follow oxidation kinetics on fast and slow timescales are described conceptually in Fig. 1. The slow-timescales 2D LLC method (Fig. 1b, c) was used in lysates and in cells, while fast oxidations were followed using the 1D method (Fig. 1d, e) in vitro.

All NMR pulse sequences used herein (Fig. 1) start with LLC excitation. LLCs are excited herein via ${\rho }_{\mathrm{LLC}}^{0}$ spin order induced using the “SLIC” method introduced by DeVience, Walsworth and Rosen^43^. They are subsequently sustained by a radio-frequency field applied with a large amplitude compared to the frequency difference between the spins (“Materials and Methods”). The LLC operator ${Q}_{{LLC}}=|{S}_{0}\rangle \langle {T}_{0}|+|{T}_{0}\rangle \langle {S}_{0}|+i(|{S}_{0}\rangle \langle {T}_{0}|-|{T}_{0}\rangle \langle {S}_{0}|)$ has a maximal projection on the initial state, and during a standard^20^ indirect-dimension *t*_1_ of 2D LLC experiments (Fig. 1b), its measured projection slowly decays while oscillating at the frequency of the *J*-coupling between spins *I* and *S*. In terms of Cartesian operators, the real component of this operator is ${Q}_{\mathrm{LLC}}^{//}={I}_{x}-\,{S}_{x}$ and the imaginary (transverse) component is ${Q}_{\mathrm{LLC}}^{\perp}=\left(2{I}_{z}{S}_{y}-2{I}_{y}{S}_{z}\right)$, ${\sigma }_{\mathrm{LLC}}\left({t}_{1}\right)=[\left(\,{I}_{x}-\,{S}_{x}\right)\cos \left(2\pi J{t}_{1}\right)+\left(2{I}_{y}{S}_{z}-2{I}_{z}{S}_{y}\right)\sin \left(2\pi J{t}_{1}\right)]\exp (-{R}_{\mathrm{LLC}}{t}_{1})$.

The obtained signal decays with the relaxation rate constant ${R}_{\mathrm{LLC}}=\frac{1}{{T}_{\mathrm{LLC}}}.\,$The *T*_LLC_ values can be significantly longer than the transverse relaxation time constants (*T*₂) of single-quantum coherences: when the intra spin-pair dipolar interaction is dominant, the ratio *T*_LLC_/*T*_2_ is larger than 3 in small molecules, can go up to a factor 9 in large molecules, and well beyond this factor in the presence of field inhomogeneities, as LLCs are immune to inhomogeneous broadening^20^. When observed in 2D spectroscopy, this oscillation results in narrow linewidths in the indirect dimension, as the transverse relaxation is improved, leading to narrowed linewidths:$\,\triangle{\mathrm{\upsilon}}_{\mathrm{LLC}}=\,{({\pi T}_{\mathrm{LLC}})}^{-1}\ll \,{\left({\pi T}_{2}\right)}^{-1}.$ For direct-dimension observation (Fig. 1d), the sustaining period is interleaved by acquisition delays, as initially conceived when LLCs were invented^20^ and then described in ref. ^46^. Multiple-windowed detection within a single experiment comes with a drawback: observed oscillations can feature reduced lifetimes due to the discontinuous sustaining. However, we observed for glutathione that the direct-dimension method for LLCs, which enables real-time detection of chemical kinetics, also preserves significant lifetimes, as detailed below.

### NMR-based observations of oxidation kinetics on the time scale of seconds

**Fig. 2:**
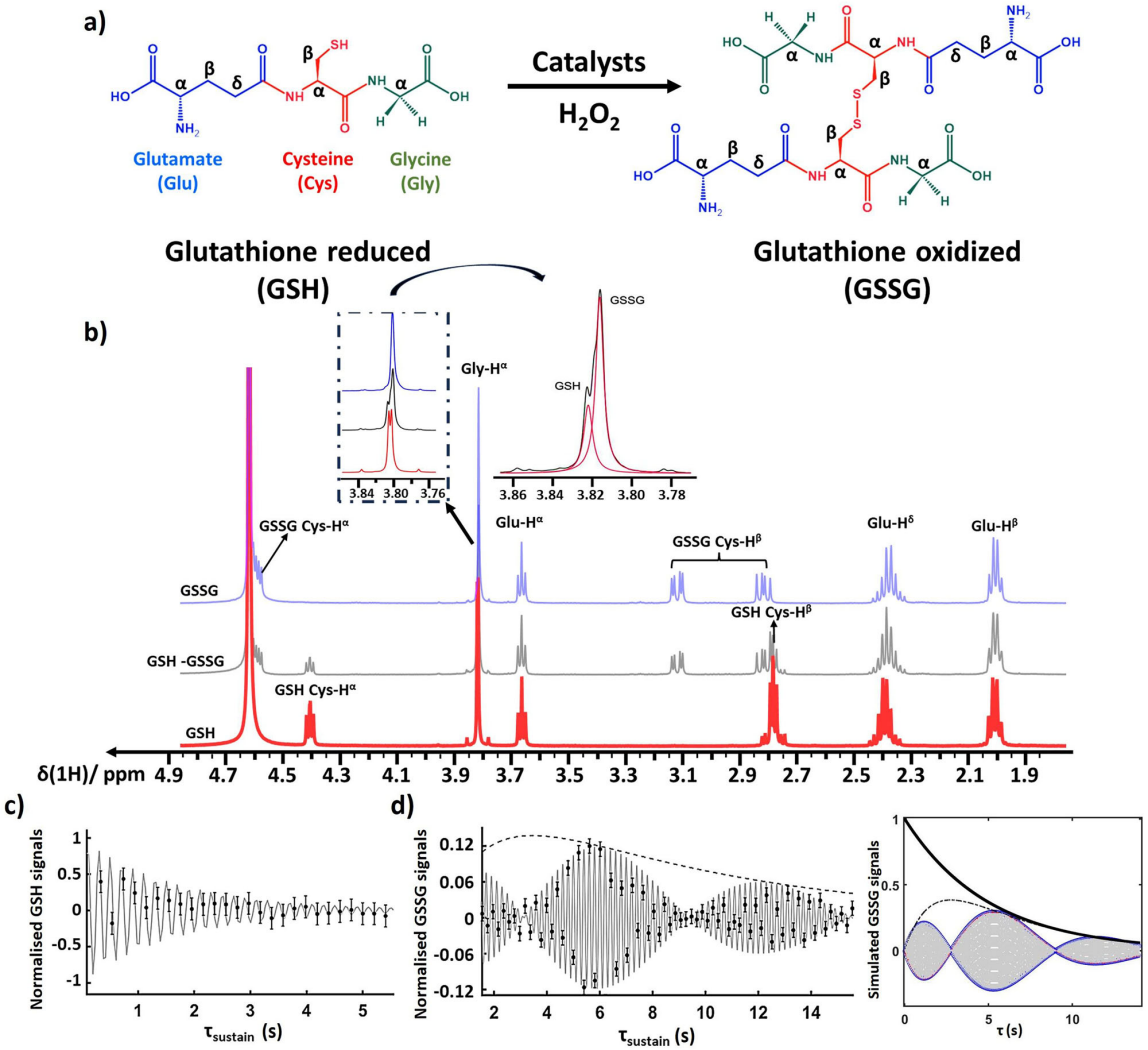
Biochemical kinetics of GSH oxidation followed by direct-dimension 1D LLCs. **a** Reaction of glutathione (GSH) with H_2_O_2_ catalyzed by iodide salts, producing glutathione disulfide (GSSG). **b** Proton 1D spectra of GSH and GSSG and insert for the region of aliphatic Gly signals (at equimolar conditions). **c** Direct detection of long-lived coherences of GSH-Gly-(H^α2^, H^α3^) (dots) during the reaction. The observed oscillation was fitted as: ${I}_{(\tau )}=\,{I}_{(0)}\exp (-{R}_{\mathrm{LLC},\mathrm{GSH}}^{\mathrm{eff},\mathrm{ox}}\tau )\cos \left(2\pi {v}_{\mathrm{GSH}}^{\mathrm{eff},\mathrm{ox}}\tau \right)$, yielding ${R}_{\mathrm{LLC},\mathrm{GSH}}^{\mathrm{eff},\mathrm{ox}}=0.6\,\pm 0.1\,{{\rm{s}}}^{-1}$ and ${v}_{\mathrm{GSH}}^{\mathrm{eff},\mathrm{ox}}=\,5.0\,\pm 0.1\,\mathrm{Hz}$. **d** The build-up of GSSG signals obtained via the reaction in A, under conditions described in “Materials and Methods”, was fitted with: ${I}_{(\tau )}=\,{I}_{(0)}\exp \left(-{R}_{\mathrm{LLC},\mathrm{GSSG}}^{\mathrm{eff},\mathrm{ox}}\tau \right)[1-\exp \left(-{k}_{\mathrm{ox}}\tau \right)]\cos \left(2\pi {v}_{\mathrm{GSSG}}^{\mathrm{eff},\mathrm{ox}}\tau \right)\cos \left(2\pi {\upsilon }^{0}\tau \right)$ yields ${R}_{\mathrm{LLC},\mathrm{GSSG}}^{\mathrm{eff},\mathrm{ox}}=0.14\,\pm 0.05\,{{\rm{s}}}^{-1}$, ${k}_{\mathrm{ox}}=0.3\,\pm 0.1\,{{\rm{s}}}^{-1}$, ${v}_{\mathrm{GSSG}}^{\mathrm{eff},\mathrm{ox}}$ $=2.5\,\pm 0.2\,$Hz and ${\upsilon }^{0}=0.08\,\pm 0.02\,$Hz. The insert shows the calculated effect induced by sampling of GSSG oscillation occurring with *J*^GSSG-Gly^ = 17.92 $\pm \,0.01\,$Hz at the sampling rate dictated by the GSH *J*-coupling, *F* = *J*^GSH-Gly^ = 17.84 $\pm 0.01\,$Hz, a slow-oscillation pattern at the value of the difference ${\upsilon }^{0}=$*J*^GSSG-Gly^-*J*^GSH-Gly^ = 0.08 $\pm \,0.02\,$Hz. This reproduces the experimentally-observed additional oscillation. Fitting methods were similar to those used previously for following GSH oxidation^13^ via single-excitation/multiple observations long-lived states (LLS) spectroscopy.

To explore the potential of LLC for the detection of fast oxidation processes, we monitored the transformation of glutathione, GSH, into its oxidized form, glutathione disulfide, GSSG (Fig. 2a). The two Glycine H^α^ protons in GSH, GSH-Gly (H^α2^, H^α3^) constitute a strongly-coupled system (Fig. 2b), described in ref. ^13^. Standard 2D LLCs were recorded for control feature signals in the indirect dimension at ${J}^{\mathrm{Gly}-({{\rm{H}}}^{\alpha 2},\,{{\rm{H}}}^{\alpha 3})}$, as expected. The oscillation was fitted, obtaining ${R}_{\mathrm{LLC}}^{\mathrm{GSH}-\mathrm{Gly}}=0.24\,\pm 0.03\,{{\rm{s}}}^{-1}$, corresponding to a decay time constant *T*_LLC_ (GSH-Gly-H^α2^, H^α3^) of circa 5 s. These LLC lifetimes are favorable for following GSH oxidation within tens of seconds and filtering GSH-Gly aliphatic signals against other metabolite proton signals, which feature transverse relaxation times below the threshold of seconds. The direct-dimension LLC sequence (Fig. 1d) based on “SLIC” excitation^43^ was used to follow fast oxidation (as described in Materials and methods): a first 90° pulse was followed by a spin-locking field with amplitude ${\upsilon }_{\mathrm{SL}}=\,{J}^{\mathrm{Gly}-({{\rm{H}}}^{\alpha 2},\,{{\rm{H}}}^{\alpha 3})}$_._ The sustaining radio-frequency was applied during delays *τ*_sustain_ interleaved with acquisition delays *τ*_off_, allowing refocusing of ${Q}_{\mathrm{LLC}}^{//}={I}_{x}-\,{S}_{x}$ via *J*-coupling evolution and, respectively, free precession of *I* and *S* spins around the carrier. LLC’s decay during *N* cycles of successive sustaining radio-frequency and acquisition delays, τ = *N*(*τ*_on_ + *τ*_off_), here extended to *τ*_max_~3*T*_LLC_ ~15 s, a delay that can be afforded while preserving observable signals. Spin dynamics simulations using Spinach libraries^47^ were used to calculate the LLC evolution under the alternate radio-frequency sustaining, and the experimental oscillating decays were reproduced with parameters adjusted to GSH-Gly aliphatic protons. The oscillation of GSH-Gly (H^α2^, H^α3^) observed in the presence of hydrogen peroxide (“Materials and methods”) via directly-detected LLCs (Fig. 2c and Supplementary Note 2) was fitted to obtain, during GSH oxidation, an effective decay rate constant ${R}_{\mathrm{LLC},\mathrm{GSH}}^{\mathrm{eff},\mathrm{ox}}=0.6\,\pm 0.1\,{{\rm{s}}}^{-1}$, faster compared to the value measured in GSH under stable conditions in the absence of hydrogen peroxide using the same 1D sequence, ${R}_{\mathrm{LLC},\mathrm{GSH}}^{\mathrm{eff}}=0.3\,\pm 0.1\,{{\rm{s}}}^{-1}$. This 1D-detected value is an average between continuously-sustained ${R}_{\mathrm{LLC},\mathrm{GSH}}$ and free-decaying ${R}_{2}^{\mathrm{GSH}-\mathrm{Gly}}$. The value of the decay rate constant during the oxidation reaction is increased ${R}_{\mathrm{LLC},\mathrm{GSH}}^{\mathrm{eff},\mathrm{ox}}$ compared to ${R}_{\mathrm{LLC},\mathrm{GSH}}^{\mathrm{eff}}$ by the contribution of the oxidation rate constant *k*_ox_: ${R}_{\mathrm{LLC},\mathrm{GSH}}^{\mathrm{eff},\mathrm{ox}}=\,{k}_{\mathrm{GSH}}^{\mathrm{ox}}+\,{R}_{\mathrm{LLC},\mathrm{GSH}}^{\mathrm{eff}}$. During the time course of the experiment, the appearance of GSSG-Gly (H^α2^, H^α3^), was observed (Fig. 2d). The GSSG build-up rate obtained from the fit, which assumes first-order GSH→GSSG conversion, is ${k}_{\mathrm{ox}}=0.3\,\pm 0.1\,{{\rm{s}}}^{-1}$. This matches, within experimental errors, the GSH-based determined difference between the effective relaxation rate during the oxidation reaction, ${k}_{\mathrm{GSH}}^{\mathrm{ox}}=\,{R}_{\mathrm{LLC},\mathrm{GSH}}^{\mathrm{eff},\mathrm{ox}}-\,{R}_{\mathrm{LLC},\mathrm{GSH}}^{\mathrm{eff}}=0.3\,\pm 0.2\,{{\rm{s}}}^{-1}$. The relaxation rate constant recorded for GSSG-Gly during conversion for GSSG-Gly-LLC was$\,{R}_{\mathrm{LLC},\mathrm{GSSG}}^{\mathrm{eff},\mathrm{ox}}=0.14\,\pm 0.05\,{{\rm{s}}}^{-1}$. Therefore, LLC lifetimes for GSSG are promising for the following oxidations in cells in real time.

### NMR detection of oxidation kinetics in cell lysates on the time scale of hours

**Fig. 3:**
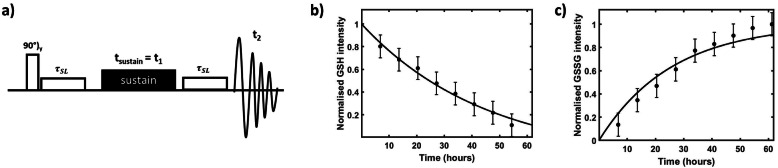
Biochemical kinetics of GSH oxidation followed by 2D LLCs. **a** 2D Long-lived coherences experiment used to observe the slow oxidation of the GSH. A refocusing SLIC step of duration *τ*_SL_ is added for in-phase detection during *t*_2_ **b** Kinetics of NMR-detected GSH concentrations during KI-catalyzed oxidation in Glioblastoma U-251 lysates. The observed signals were fitted as: ${C}_{(T)}^{\mathrm{GSH}}/{C}_{(0)}^{\mathrm{GSH}}=\,\exp (-{k}_{\mathrm{ox},\mathrm{GSH}}^{\mathrm{slow}}T)$ yielding ${k}_{\mathrm{ox},\mathrm{GSH}}^{\mathrm{slow}}\,$= 0.022 $\pm$ 0.003 h^−1^. **c** Kinetics of NMR-detected GSSG concentrations during oxidation (build-up of the oxidized form) in Glioblastoma U-251 lysates. The observed signals were fitted as: ${C}_{(T)}^{\mathrm{GSSG}}/{C}_{(0)}^{\mathrm{GSSG}}=1-\exp (-{k}_{\mathrm{ox},\mathrm{GSSG}}^{\mathrm{slow}}T)$ yielding ${k}_{\mathrm{ox},\mathrm{GSSG}}^{\mathrm{slow}}$ = 0.039 $\pm$ 0.007 h^−1^.

In response to most types of naturally-occurring oxidative stress -with the notable exception of FLASH radiation—the time scale of GSH oxidation kinetics in cells is slow, occurring generally on timescales of hours. We followed slow GSH oxidation induced using low quantities of oxidant and catalyst (Fig. 3, “Materials and Methods”). In order to extract the slow kinetics rate, ${k}_{\mathrm{ox}}^{\mathrm{slow}}$, a pair of experiments was used: a long 2D-LLC experiment and a standard 90°-excitation-detection 1D NMR experiment, both launched every 7 h. The long experimental time allowed us to extend the LLC pulse sequence: it was modified in order to observe in-phase (positive) GSH and GSSG signals, easier to process and use for quantitative determination. For this, a continuous spin-lock period equal to the one used for LLC excitation and applied on resonance with the nearly-equivalent spins at a nutation frequency set to the value of ${J}^{\mathrm{GSH},\mathrm{Gly}-({{\rm{H}}}^{\alpha 2},\,{{\rm{H}}}^{\alpha 3})}$ was added after the radio-frequency irradiation (Fig. 3a)^43^. The 2D-LLC experiment yielded a difference of 0.10 $\pm$ 0.08 Hz between the GSH and GSSG *J*-coupling values of aliphatic Gly protons in the indirect dimension, in addition to the direct-dimension frequency separation of 15 Hz. We observed the consumption of GSH via the GSH-Gly-H^α^ central signal and the appearance of GSSG via the GSSG-Gly-H^α^ central signal. The kinetics was modeled as first order, compatible with the concentrations of H_2_O_2_ introduced.

An oxidation rate value ${k}_{\mathrm{ox},\mathrm{GSH}}^{\mathrm{slow}}\,$= 0.022 $\pm$ 0.003 h^−1^ was obtained from the GSH decay (Fig. 3b), and a similar value was determined, within the measurement error, from the GSSG build-up (Fig. 3c). In order to confirm these values, we have equally followed another aliphatic proton signal in the two forms, the Cys-H^α^ signal. The analysis of kinetics using this signal yields ${k}_{\mathrm{ox},\mathrm{Cys}}^{\mathrm{slow}}$ = 0.031 $\pm$ 0.002 h^−1^, a value which is similar to the one obtained using the Gly signals above (within Monte-Carlo-based errors), thus confirming the results.

### GSH detection in cells

**Fig. 4:**
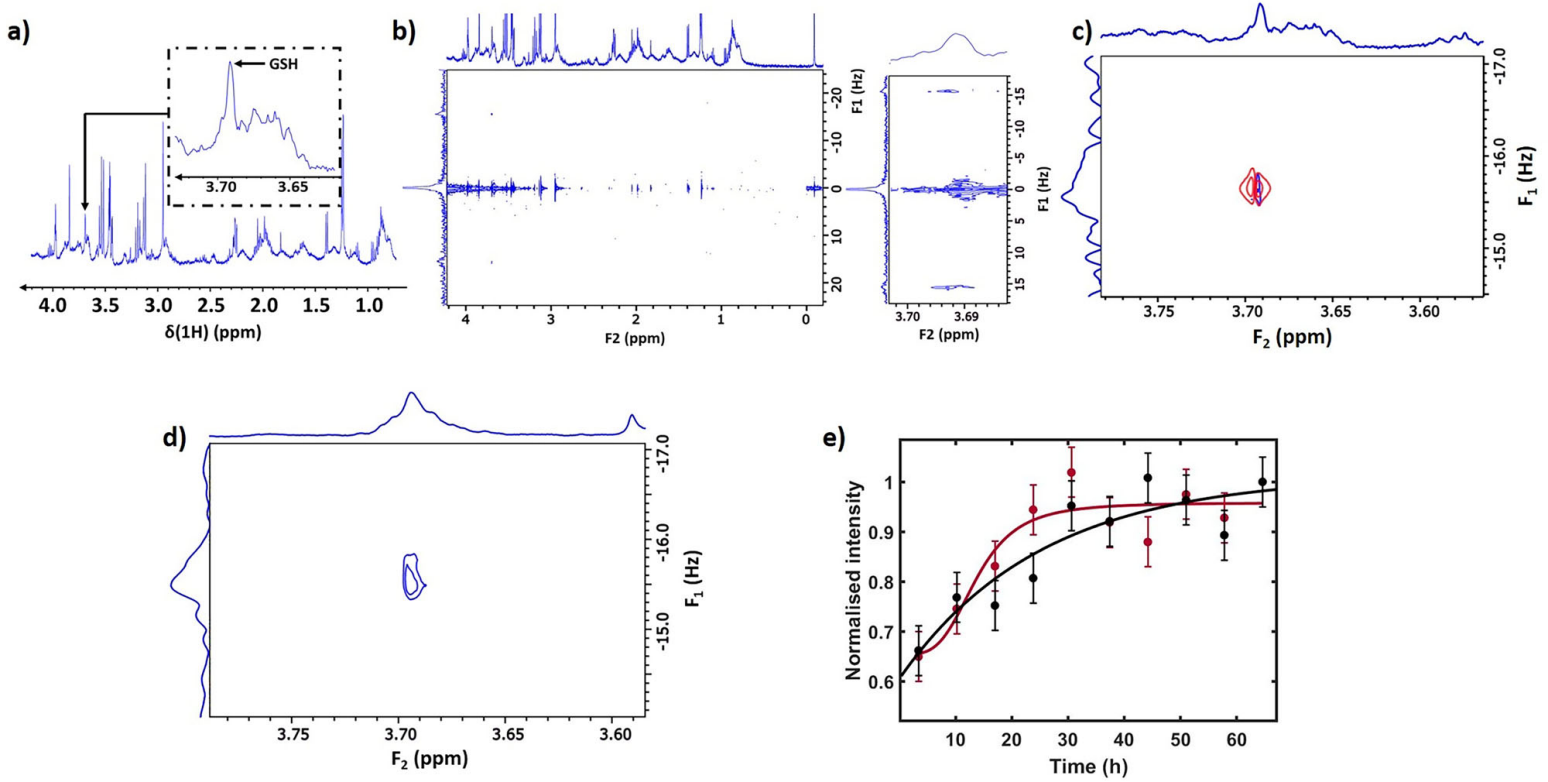
GSH detection in cells. **a** 1D NMR spectrum of glioblastoma cells; a zoom of the region of aliphatic GSH-Gly signals is shown on top; **b** 2D-LLC spectrum recorded in RAJI-type lymphoblastoid cells. Resonances of GSH-Gly-(H^α2^, H^α3^) are shown in the zoom; **c** Comparison of 2D LLC signals recorded on a sample of GSH in natural abundance in glioblastoma U-251 (blue) and on GSH-incubated glioblastoma lysates (red). **d** 2D LLC spectrum recorded in RAJI-type lymphoblastoid cells. Resonances of GSH-Gly-(H^α2^, H^α3^) are outlined in the zoom; **e** Kinetics of intracellular NMR-detected concentrations of GSH (red) and GSSG (black) in RAMOS-type lymphoblastoid cells that were incubated exogenously with GSH 24 h before the start of 2D-LLC recording (Supporting Information). The data shows that GSH concentrations increase until they reach a plateau, and oxidized glutathione GSSG production follows that of GSH with an estimated half-time *T*_GSSG_ = 25 h for the build-up.

The 2D LLC’s experiment was used for GSH detection in cell lysates. The challenge is to filter GSH and GSSG signals from the signals of other metabolites (Fig. 4a). In U-251 MG human brain glioblastoma cell lysates, GSH was identified in natural abundance via Gly-(H^α2^,H^α3^) signals at 3.68 ppm (Fig. 4a).

The reference 2D-LLC experiment performed on lysates yielded the GSH-Gly signal in Fig. 4b. In order to confirm this, we added (“spiked”) 1 mM GSH inside the sample and recorded another 2D LLC experiment. The same signal was observed (Fig. 4c).

The GSH-Gly signal was subsequently detected by 2D-LLC in natural concentration in immune RAJI-type cells (Fig. 4d). For inducing de novo GSH production in RAMOS cells^48^, one can use cell irradiation—herein, cells were irradiated with a dose of ca 10 Gy using high-dose-rate electrons stemming from a high-power laser accelerator^2^—or via incubation with GSH, which induces a positive feedback response. We have followed the time course of GSH/GSSG signals in the pre-incubated cells via 2D-LLC (Fig. 4e and Supplementary Note 3). The experimentally-detected 2D-LLC data increase to saturation within the time window of 24–55 h post cell treatment (Fig. 4e), as NMR recordings start 24 h post induction. At variance with lysates, the in-cell formation of GSSG is due to contributions from both pre-existing and induced glutathione; therefore, only the estimated build-up half-time *T*_GSSG_ was derived, rather than a characteristic time. The biochemical detection of GSH-producing enzyme Glutamyl Cysteine Ligase (GCL) highlighted the Ramos B lymphocytes treatment response (Supporting Material) as a 7–8 fold increase in the rate-limiting GCL enzyme precursor, which explains the observed surge in GSH production in these cells by NMR. Thus, biochemical experiments confirmed the 2D-LLC data.

## Conclusions

We followed by LLC NMR spectroscopy in real time the oxidation kinetics of GSH, the most abundant antioxidant in fast-proliferating cells, on timescales of tens of seconds and of several hours. The 1D WINDOW-LLC method enables the follow-up of fast redox reactions in the presence of high fluxes of oxidizing agents, as triggered in FLASH radiobiology using high dose-rate radiation. The 2D-LLC method is useful for following slow oxidations in cells. LLC ^1^H-based methods are thus added to the repertoire of methods relying on stable-isotope labeling for detecting biomarker transformations against the metabolic cell background. These LLC approaches may also be useful for the development of functional MRI.

## Materials and methods

### NMR hardware, operating conditions, and software used for processing

NMR experiments were conducted on Bruker Avance III spectrometers, operating at 500 MHz proton Larmor frequency, equipped with a 5 mm radio-frequency probes. The temperature was set at *T* = 25 °C. NMR spectra were acquired using TopSpin® and spectral intensities extracted using Topspin® and Matlab functions. Errors in spectral intensities are determined by the spectral noise, and the confidence ranges for fitted kinetic constants are based on 1000 Monte–Carlo variations of experimental points within experimental errors, yielding the reported standard deviations of fitted constants.

### GSH oxidation

For fast GSH oxidation, 5 mg of GSH was dissolved in 450 μL D_2_O. We added 50 μL of KI solution at 55 mM and 50 μL H_2_O_2_ (30%). The temperature was set at *T* = 25 °C. The carrier was set in the middle of the two GSH-Gly-(H^α2^, H^α3^) resonances, i.e., Δ*ν*_RF_ = 0. The 1D LLC pulse sequence starts with a hard π/2 pulse followed by a square-amplitude adiabatic inversion pulse with amplitude *ν*_SL_ = 17.8 Hz of duration τ_SL_ = 59 ms for LLC excitation. The continuous-wave sustaining amplitude was *ν*_1_ = 4 kHz. LLCs were sustained using delays ${\tau }_{\mathrm{on}}\,$= 28 ms and ${\tau }_{\mathrm{off}}$ = 157 ms, optimized by numerical simulations (Supplementary Note 4) to afford refocusing of the coherence ${Q}_{\mathrm{LLC}}^{//}$ at the end of each interval while the oscillation proceeds. NMR spectra were acquired using TopSpin and spectral intensities extracted using Topspin and Matlab functions.

### Cell lysates and NMR experiments in lysates

U-251 MG human brain glioblastoma cells (CLS Cell Line Service, Eppelheim, Germany) were cultured in Dulbecco’s Modified Eagle Medium (DMEM) supplemented with 10% fetal bovine serum and 0.1% Penicillin–Streptomycin (Biowest, Nuaillé, France). Cells were seeded in 25 mL flasks (TPP Techno Plastic Products AG, Trasadingen, Switzerland) at an amount of approximately 2,400,000 cells/flask and incubated in standard conditions of temperature and humidity for 24 h before irradiation to allow their attachment. Glioblastoma cells were washed three times with ice-cold PBS (phosphate buffer saline) for a few minutes and detached using 1% Trypsin in PBS. Cell samples were stored deep-frozen for the upcoming lysis and metabolite extraction procedure. On the day of the NMR experiment, the cell pellets were brought to ambient temperature and suspended in an ice-cold mixture of acetonitrile and deionized water at 1:1 volume ratio, and lysed using an ultrasound probe. The lysates were then centrifuged at 4 °C. The supernatant was carefully collected, and the solvent was evaporated using a rotoevaporator. For the NMR experiments, the dry residue was dissolved in D_2_O containing 1 M phosphate buffer at pH = 7.0.

Proton spectra were recorded using a water suppression pulse program^49,50^. A repetition time of 1.6 s was used for recovery of longitudinal magnetization between scans, and a number of 128 accumulations was set, resulting in a total measurement time of 14 min. Sample temperature was maintained at 25 °C during measurements.

For slow GSH oxidation, 1 mM of GSH was added in lysates. We added 20 μL of KI solution at 0.02 mM concentration dissolved in H_2_O_2_ (0.01%). Proton spectra were recorded using a single pulse program^49,50^ (total measurement time of 1 min/experiment). Sample temperature was maintained at 25 °C during measurements. The NMR spectra were processed and analyzed using Topspin and Matlab. 2D LLC experiments were recorded with 8 transients and a recovery delay of 2.6 s in the direct dimension. The carrier was set in the middle of the two GSH-Gly(H^α2,^H^α3^) resonances. The LLC pulse sequence in Fig. 1b was used, with a “SLIC” excitation step as described above. Sustaining using continuous-wave irradiation was performed with an amplitude of 4 kHz during the variable indirect-dimension times, *t*_1_.

2D LLC reference experiments were recorded with 8 transients and a recovery delay of 5 s in the direct dimension. A number of 512 points was recorded in the indirect dimension, and time-proportional phase incrementation (TPPI) was used for phase-sensitive detection. The continuous-wave irradiation was set in the middle of the two GSH-Gly-(H^α2^, H^α3^) resonances with an amplitude of 4 kHz. All other parameters were set as above.

### Cell cultures, biochemical characterization, and 2D LLC experiments in cells

Cells from either Raji or Ramos lines of lymphoblastoid Burkitt’s lymphoma human cells were grown in suspension in RPMI medium (Gibco) supplemented with 10% FBS, 0.1% Penicillin–Streptomycin, to a density of 6 × 10^5^ cells/ml in two 50 ml flasks, at 37 °C and 5% CO_2_. At this density, for each cell type, the medium of one flask is supplemented with 5 ml of 50 mM solution of reduced form GSH (final concentration 5 mM GSH), whereas to the control flask only 5 ml of plain medium was added. Cells from both flasks were grown for 24 more hours under the same conditions. At the end, individually-harvested cells were spun at 500 rpm for 10 min, the pellets were washed in PBS, respun, and for each lot, 5 × 10^6^ cells/pellet were resuspended in 500 μl D_2_O for each recording. RAMOS cells from two batches (±GSH treatment) were subjected to high-dose-rate electron beam irradiation (IR) stemming from a high-power laser (1 PW) interaction with a gas target (50 pulses @ 0.1 Gy/ns, cumulative dose of ca 10 Gy). A sample of 5 × 10^6^ cells from each treatment at each recorded time was analyzed by NMR. 2D LLC experiments were recorded with 8 transients and a recovery delay of 5 s in the direct dimension. All other parameters were set as above. For the western blot, the chemiluminescent detection scan image of the membrane incubated with anti-GCLM antibody (Sigma-Aldrich 1D19), resulting from the electrophoretic separation of the transferred 10% SDS-PAGE gels (cell extracts correspond to 3 × 10^5^cells/well). The densitometric quantifications (expressed as ratios of GSH stimulated versus non-stimulated cells GSH+/GSH−) of the specifically detected bands (indicated by arrows) are obtained using Image-Lab software (Bio-Rad) and are shown as histograms.

### Reporting summary

Further information on research design is available in the Nature Portfolio Reporting Summary linked to this article.

## Supplementary information

Supplementary Information

Reporting Summary

## Data Availability

Data acquired during this study are available at https://biophysicsmr.wordpress.com/data-llc_2025/ and 10.6084/m9.figshare.31028896 (activated when the article is available online).

## Code availability

The software used for simulations is available in the supplementary information and in the online archive mentioned above.
