# Supplementary material for: Long-lived coherences for the observation of oxidation kinetics on different timescales by NMR
Source: Commun Chem. 2026 Apr 16;9:211. doi: 10.1038/s42004-026-02002-w (PMC13269996; doi:10.1038/s42004-026-02002-w)
Supplement: Supplementary file 1 — Supplementary Information [file 42004_2026_2002_MOESM1_ESM.pdf]

Supporting Information

Table of Contents

1. Comparison table for relaxation rate constants for long-lived coherences and transverse coherences detected in 1D experiments: table of measured relaxation rate constants in GSH / GSSG

2. Glutathione GSH and GSSG identification in fast kinetics in vitro

3. Quantification of glutathione *in cells;* Agreement between 2D-LLC and biochemical detection of *de novo* GSH synthesis

4. Program used for simulations in Spinach under Matlab

1. **Supplementary Note 1:**

Supplementary table 1: *Table of measured relaxation rate constants in GSH / GSSG*

	**Conditions**	**Relaxation rate value**
R_{LLC}^{GSH}	LLC classical method	0.24 ± 0.03 s^{-1}
$R_2{}^{eff}{}_{,GSH}$	Transverse relaxation rate, windowed	0.9 ± 0.2 s^{-1}
$R_{LLC,GSH}^{eff}$	Windowed-1D LLC without oxidation	0.3 +/- 0.1 s^{-1}
$R_{LLC,GSH}^{eff,ox}$	Windowed-1D LLC in the presence of H_2O_2 (conditions in *Materials and Methods*)	0.6 +/- 0.1 s^{-1}
$R_{LLC,GSSG}^{eff,ox}$	Windowed-1D LLC GSSG	0.14 +/- 0.05 s^{-1}
k_{ox}	Windowed-1D LLC in the presence of H_2O_2 (conditions in *Materials and Methods*)	0.3 +/- 0.1 s^{-1}
	Slow kinetics detected via 2D LLC	
$k_{GSH}^{eff,ox}$	2D LLC in D_2O detected via GSH decay	0.07 +/- 0.02 h^{-1}
$k_{GSSG}^{eff,ox}$	2D LLC in D_2O detected via GSSG build-up	0.09 +/- 0.02 h^{-1}
$k_{GSH}^{eff,ox}$	2D LLC with H_2O_2 in glioblastoma lysates detected via GSH decay	0.022 +/- 0.003 h^{-1}
$k_{GSSG}^{eff,ox}$	2D LLC with H_2O_2 in glioblastoma lysates detected via GSSG build-up	0.039 +/- 0.01 h^{-1}

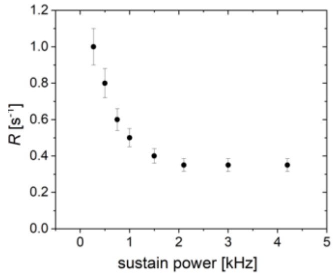

Supplementary Figure 1: Window-LLC relaxation rate constants obtained from the single-shot LLC experiment series as a function of the amplitude of the sustaining radio-frequency field.

2. Supplementary Note 2:

Fast kinetics in cell lysates

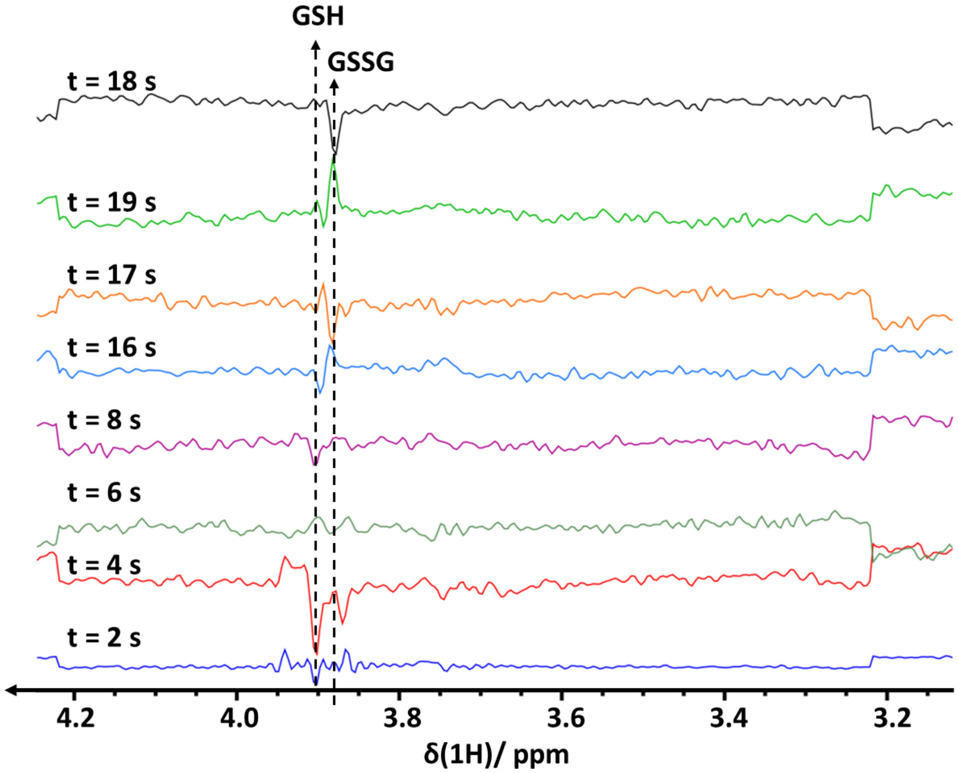

Supplementary Figure 2: 1D LLC experiments in fast kinetics. Sequential 1D spectra recorded at various time points during the rapid oxidation of GSH: t = 2 s (blue), t = 4 s (red), t = 6 s (green), t = 8 s (purple), t = 16 s (dark blue), t = 17 s (orange), t = 18 s (black), and t = 19 s (dark green).

3 Supplementary note 3

Detection of glutathione production in cells.

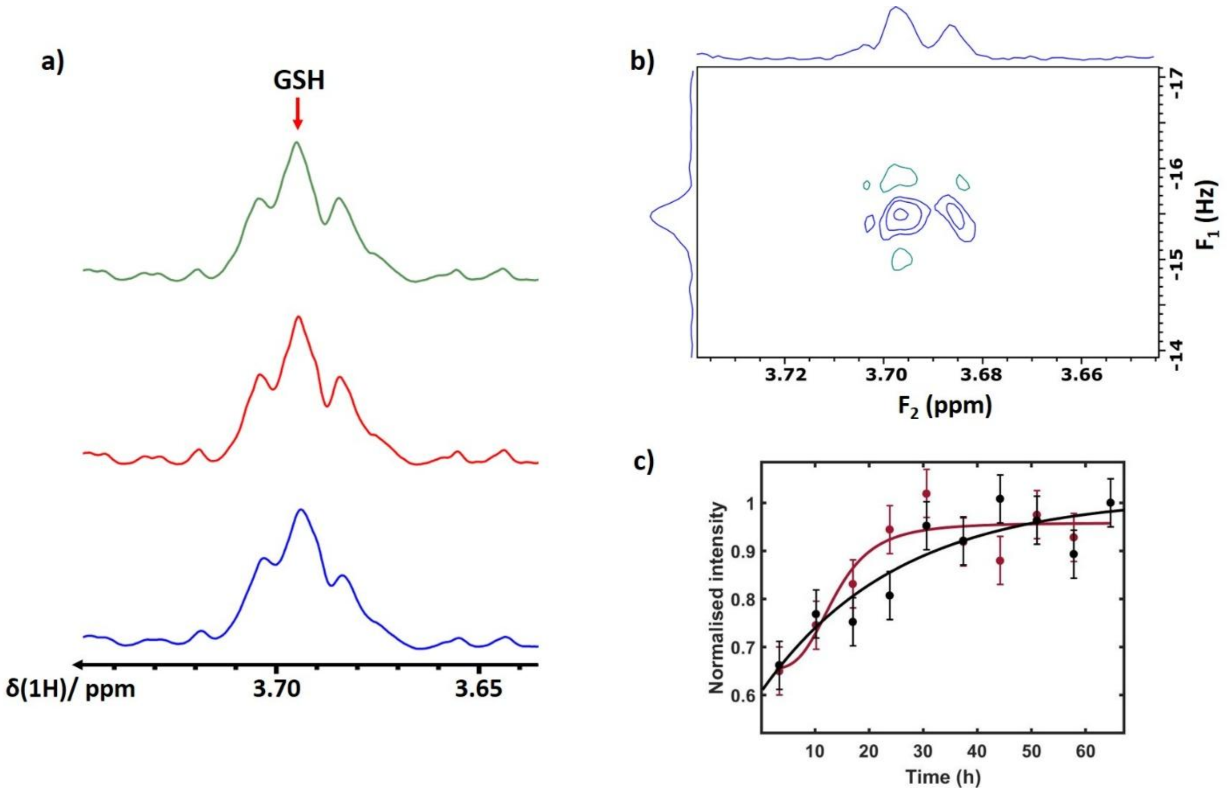

Supplementary Figure 3: a) Overlay of 1D spectra in RAMOS cells in the presence of incubated GSH (blue at t = 0 h, red at t = 30 h, and green at t = 60 h). b) 2D spectrum at t=0 h obtained in RAMOS cells. c) 2D-LLC inferred time-dependent changes in concentrations detected via the aliphatic protons of GSH-Gly (red) and GSSG-Gly (black) during KI-catalyzed oxidation in RAMOS cells. The data for GSSG formation in time (T) show that it reaches a plateau within this time window and GSSG production can be fitted as: $C_{(T)}^{GSSG}/C_{(0)}^{GSSG} = I(T) = I_0 + A_0\left(1 - \exp\left(-k_{ox}^{slow,\,GSSG}T\right)\right)$, with $k_{ox}^{slow,GSSG}$ = 0.04 ± 0.01 h^{-1} for GSSG formation.

Agreement between 2D-LLC and biochemical detection of *de novo* GSH synthesis induced in RAMOS cells, in the presence/absence of oxidative stress conditions caused by irradiation.

A way of inducing *de novo* GSH production in RAMOS cells (White 2003, doi:10.1016/S0003-2697(03)00143-X) is incubation with GSH, which induces a positive feedback response. After growing and GSH treatment (see main manuscript, Materials and Methods) RAMOS cells from two batches (+/- GSH treatment) were subjected to high dose-rate electron beam irradiation (IR) stemming from a high-power laser (1 PW) interaction with a gas target (50 pulses @ 0.1 Gy/ns, cumulative dose of 5 Gy). A sample of 5x10^6 cells from each treatment at each recorded time was analyzed by NMR and data are shown in Fig. 4E. (main manuscript), identifying in their spectra either GSH or its oxidation product GSSG signature (initiation in 2D LLC data corresponds with 24 hours post treatment). In parallel, we chose to

detect biochemically by Western Immunoblot both qualitatively and quantitatively how GSH / IR treatments affect the synthesis of the subunits of Glutamyl Cysteine Ligase (GCL). GCL catalyzes the rate-limiting step in GSH synthesis (the first step in GSH synthesis the formation of the gamma glutamyl cysteine dipeptide) and its presence is prerequisite for glutathione synthesis (White 2003, doi:10.1016/S0003-2697(03)00143-X). GCL is a heterodimer made of a catalytic subunit (GCLC ~ 72 kDa) and a modifier subunit (GCLM~ 31 kDa). The modifier subunit of GCL enzyme, GCLM, which controls the rate-limiting step in GSH synthesis, was identified as the key component of *de novo* GSH synthesis (Figure).

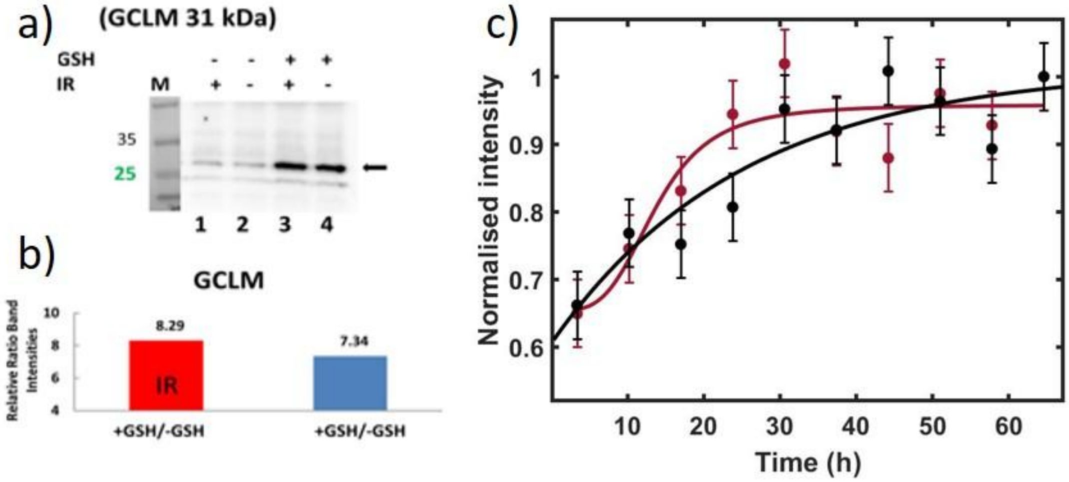

Supplementary Figure 4: Detection of cellular components leading to GSH formation by 2D-LLC and Western Immunoblot a) The 'modifier' constitutive subunit of glutamate-cysteine ligase (GCLM), the first-expressed enzyme regulating GSH build-up in cells, from Ramos cell extracts. In A cells were either untreated (lanes 1 and 2) or treated (lanes 3 and 4) with 5mM GSH for 24 hours prior to +/- exposure to 5Gy irradiation with e- (lanes 1 and 3 irradiated cells, whereas lanes 2 and 4 from non-irradiated cells).
b) Histogram heights show ratios of densitometric quantification values of GCLM from cells treated with GSH versus values of nontreated cells from figure A; in red (IR, irradiated cells), in blue non-irradiated cells.
c) 2D-LLC detection of GSH and GSSG build-up as a function of time in the same RAMOS cells as above (samples of ca 5x10^6 cells/ NMR tube).

Figure (A) shows the chemiluminescent detection scan image of the membrane incubated with anti-GCLM antibody (Sigma-Aldrich 1D19), resulting after the electrophoretic separation of the transferred 10% SDS-PAGE gels (cell extracts correspond to 3 x10^5cells/well). The densitometric quantifications (expressed as ratios of GSH stimulated versus non-stimulated cells GSH+/GSH-) of the specifically detected bands (indicated by arrows) are obtained using Image-Lab software (Bio-Rad) and are shown as histograms.

GSH treatment induces in cells the modifier subunit at 24 h (accentuated bands in wells 3 and 4 in Figure show the GCLM component more pronounced than in wells 1 and 2 of the same figure). The discussed effect is emphasized by a detected 7-fold GCLM increase after 24 hours GSH treatment, as shown in the histograms in B. This result indicates that exogenous GSH positively feedbacks and reinforces the production of intracellular GCLM, which in turn enhances the rate of the first step of GSH synthesis. Irradiation generates in this time frame (24 hours) just a slight increase by ca 15 % in GCLM (from 7.34-fold to 8.29-fold), consistent with a potentiation of the afore-mentioned induction. Although our biochemical experiment highlights only an early Ramos B lymphocytes GSH treatment response (after the first 24 hours), this increase in GCL enzyme precursors explains why one would expect a surge in endogenous GSH production in these cells at later times. The experimentally-detected data increase to saturation in GSH and GSSG, as documented by the 2D-LLC data from these cells (Figure, C) within 24-55 hrs. time window (ascending slope) post GSH treatment (NMR recordings start delayed with 24 hrs. post GSH addition). The saturation of GSH production and its 2D-LLC kinetics sigmoidal saturable fit (Figure, C) strongly suggests an enzymatic induction effect caused by the exogenously added GSH. The generation of GSSG is caused by intracellular oxidation of the former in cellular processes. In conclusion, RAMOS cells stimulation leads to *de novo* endogenous GSH synthesis under normal and oxidative stress conditions (induced by e- irradiation in Gy/ns high dose-rate pulses).

Cells for immunoblot analysis were grown and treated under identical conditions with those subjected to NMR measurements, described at Matherials Methods main manuscript 2D LLC experiments in cells. Western Immunoblot methodology was done according to the Bio-Rad protocol (https://www.bio-rad.com › lsr › Bulletin_6376) described in bulletin 6376. The electrophoretic separation of the of cell lysate extracts was done in 10% SDS-PAGE gels. All gel wells were loaded with lysate from the same number of cells (3 x 10^5). The gel separated protein components were transferred onto pre-treated PVDF membranes which after blocking were incubated with specific antibodies. We were using for the first antibody a 1 : 500 dilution(anti-GCLM, 1D19, and anti-GCLC, SAB5700670 antibodies both commercial from Sigma-Aldrich), and for the secondary HRP conjugated antibody(G-21234 Thermo Fisher) a 1: 2000 dilution. Visualization was obtained with Max ECL (Enhanced Chemiluminescence Bio-Rad)solution and scanning of the developed membranes was done with ChemiDoc Scanner for final imaging. The chemiluminescent scanning images of the membranes were subjected to quantitative measurements using gray-scale densitometric band quantifications with Image-Lab software (Bio-Rad).

4. Supplementary note 4:

LLC-based NMR detection of oxidation reactions: spin-dynamics calculation for GSH oxidation to GSSG

A spin dynamics calculation of the conversion of LLC's on a generic spin-1/2 system (I,S) with *J*-coupled spins to LLC's of a form (K,R) was performed in Spinach (http://dx.doi.org/10.1016/j.jmr.2010.11.008) under Matlab. The program includes the effects of the oxidation with rate constant $k_{ox.}$. For GSH-Gly (I,S) = ($H^{\alpha 2}$, $H^{\alpha 3}$) → GSSG-Gly ($H^{\alpha 2}$, $H^{\alpha 3}$). Dipolar interactions are included for each spin pair, corresponding to chosen interaction distances d_{IS} = d_{KR} = 1.9 Å and a rotational correlation time τ_c = 50 ps. Cartesian coordinates of every spin are {[-dIS / 2, 0, 0], [dIS / 2, 0, 0], [d, -dKR/2, 0], [d, dKR/2, 0], where d is chosen d = 20 Å to exclude inter-pair dipolar interactions.

```
% ==========================================================
%           SPIN SYSTEM DEFINITION (4 PROTONS)
% First two spins:   GSH molecule
% Last two spins:    GSSG molecule
% function test_sim()
close all;
clear all;
path(path,'d:\Prog_Sim_AS\Paul\Ensemble_prog_matlab');
% ==========================================================
sys.magnet = 11.74;                    % B0 magnetic field (Tesla)
sys.isotopes = {'1H', '1H', '1H', '1H'}; % Four protons
% Interproton distances (in Å) for dipolar couplings
dIS = 1.9;
dKR = 1.9;
rot_corr_time = 50e-12;  % rotational correlation time (50 ps)

% J-couplings for the two molecules
JIS = 17.84;     % J-coupling between spins 1–2 (GSH)
JKR = 17.92;     % J-coupling between spins 3–4 (GSSG)

% Chemical shifts (in ppm-like scalar format)
inter.zeeman.scalar = {-0.0068, 0.0068, 0.026, 0.036};

% J-coupling matrix
inter.coupling.scalar{1, 2} = JIS;
inter.coupling.scalar{3, 4} = JKR;
inter.coupling.scalar{4, 4} = 0;

inter.coordinates = {[-dIS/2,0,0], [dIS/2,0,0], ...
                 [20,-dKR/2,0], [20,dKR/2,0]};


inter.chem.parts = {[1 2], [3 4]};

% Chemical exchange rate matrix
% Forward exchange from molecule 1?2 at 0.4 Hz
inter.chem.rates = [ -0.4  0;
                  0.4  0];

% Initial concentrations of the two species
inter.chem.concs = [300, 0];

% ==========================================================
%               BASIS & RELAXATION SETUP
% ==========================================================
bas.formalism = 'sphten-liouv';
bas.approximation = 'none';

inter.relaxation = {'redfield'};
inter.equilibrium = 'zero';
inter.rlx_keep   = 'secular';
inter.tau_c      = {rot_corr_time, rot_corr_time};
```

```
% Larmor frequency difference (Hz)
niuIS = (inter.zeeman.scalar(2) - inter.zeeman.scalar(1))*500;

t_irrad = 0.028;                 % CW irradiation period
t_free  = 0.0524;     % free evolution time
t_dw    = t_free / 1000;                  % dwell time


spin_system = create(sys, inter);
spin_system = basis(spin_system, bas);
spin_system = assume(spin_system, 'nmr');

% Spin raising operators
Ip = operator(spin_system, 'L+', 1);
Sp = operator(spin_system, 'L+', 2);
Kp = operator(spin_system, 'L+', 3);
Rp = operator(spin_system, 'L+', 4);

% Composite two-spin operator for GSH
ISp = operator(spin_system, 'L+', [1 2]);
ISx = (ISp + ISp') / 2;
ISy = (ISp - ISp') / (2i);

% Single-spin Cartesian operators
Ix = (Ip + Ip')/2;
Sx = (Sp + Sp')/2;
Iy = (Ip - i*Ip')/2;
Sy = (Sp - i*Sp')/2;
Kx = (Kp + Kp')/2;
Rx = (Rp + Rp')/2;
Ky = (Kp - i*Kp')/2;
Ry = (Rp - i*Rp')/2;

% Hamiltonian, Relaxation, Kinetics
H = hamiltonian(spin_system, 'comm');
R = relaxation(spin_system);
K = kinetics(spin_system);

% Total Liouvillian
L = H + 1i * R + 1i * K;

% Detection coil (transverse 1H magnetization)
coil = state(spin_system, 'L+', '1H');

% Thermal equilibrium state (Lz on spins 1 & 2)
rho0 = state(spin_system, 'Lz',[1 2]);

% ============================================================
%                EXPERIMENT SETUP
% ============================================================
amp_CW = 4000;              % CW irradiation amplitude (Hz)
t_total = t_irrad + t_free;
t_detection = t_free;

% Number of points in detection and irradiation blocks
np_detection = round(t_detection/t_dw) * 2;
np_cw = round(t_irrad/t_dw);

% Apply a 180° pulse around y
cur_state = step(spin_system, Iy, rho0, 180*pi/180);

% Then a 90° x-pulse on all spins
cur_state = step(spin_system, ...
              (operator(spin_system, 'L+', 'all') - ...
               i*operator(spin_system, 'L-', 'all'))/2, ...
               cur_state, 90*pi/180);
```

```
% Sum of all x magnetizations
sum_allX = (operator(spin_system,'L+','all') + ...
           operator(spin_system,'L-','all'))/2;

rho1z = state(spin_system, 'Lz', 1);
rho2z = state(spin_system, 'Lz', 2);
rho3z = state(spin_system, 'Lz', 3);

% Rotate z  states
rho1x = step(spin_system, Iy, rho1z, 90*pi/180);
rho2x = step(spin_system, Sy, rho2z, 90*pi/180);
rho3x = step(spin_system, Ky, rho3z, 90*pi/180);

% LLC observable: difference between anti-phase operators
rho_llc_ch = (state(spin_system, 'L+', 1) - state(spin_system, 'L-', 1))/2 ...
           -(state(spin_system, 'L+', 2) - state(spin_system, 'L-', 2))/2;

% ============================================================
%            MAIN MULTI-CYCLE SIMULATION LOOP
% ============================================================
num_cycles = 120;
timev = [];
llctime = [];
signals = [];
llcgsh_sum = [];
llcgssg_sum = [];
llctime_sum = [];

for cycle = 1:num_cycles
   % Free evolution block — used to normalize
   free_ev_norm = evolution(spin_system, L, [], cur_state, ...
                       t_dw, int64(np_detection/2)-1, 'trajectory');

   % GSH X magnetization (normalized)
   llcgsh_sum_norm = abs(sum(rho1x' * free_ev_norm));

   % CW irradiation block (state updated)
   cw_ev = evolution(spin_system, L + 2*pi*amp_CW*sum_allX, [], ...
                 cur_state, t_dw, np_cw-1, 'final');

   cur_state = cw_ev(:, end);

   % Detection block
   free_ev = evolution(spin_system, L, [], cur_state, ...
                  t_dw, int64(np_detection/2)-1, 'trajectory');

   cur_state = free_ev(:, end);

   % Detected NMR signal
   signal = coil' * free_ev;

   % Time vector assembly for all cycles
   timeadd = cycle * t_irrad * ones(1, np_detection/2);
   timenow = cycle*((np_detection/2)-1)*t_dw : ...
           t_dw : ...
           (cycle+1)*((np_detection/2)-1)*t_dw;

   timev = [timev, (timeadd + timenow)];

   % LLC observable time trace
   llctime = [llctime, rho_llc_ch' * free_ev];

   % Summed magnetizations for tracking
   llcgsh_sum = [llcgsh_sum, sum(rho1x' * free_ev)];
   llcgssg_sum = [llcgssg_sum, sum(rho3x' * free_ev)];

   signals = [signals; signal];

end
```

```
% ===========================================================
%      LLC SIGNAL — REAL / IMAG / AMPLITUDE ENVELOPE
% ===========================================================

% Compute amplitude envelope (magnitude of complex LLC)
% llc_amp = abs(llctime);   % sqrt(Re^2 + Im^2)

figure(1); clf;

plot(timev, real(llctime), 'b-', 'LineWidth', 1.8);
ylabel('LLC', 'FontSize', 14);
title('Time / s', 'FontSize', 16);
grid on;
set(gca, 'FontSize', 12, 'LineWidth', 1.2, 'Box', 'on');

%% -------- SAVE FIGURE --------
set(gcf, 'Color', 'w');
exportgraphics(gcf, 'LLC_.png', 'Resolution', 300);
savefig('LLC_.fig');

save wkspbckp;
```
